# Consistency of on-the-job training implementation for subcutaneous depot medroxyprogesterone acetate in Ghana and associated healthcare worker knowledge transfer: A cross-sectional mixed-methods study

**DOI:** 10.1371/journal.pgph.0004799

**Published:** 2026-04-30

**Authors:** Chelsey Porter Erlank, Mensimah Bentsi-Enchill, Samuel Tagoe, Joyce Ami Amedoe, Melinda Stanley, Claudette Ahliba Diogo, Kofi Issah

**Affiliations:** 1 Analytics and Implementation Research Team, Clinton Health Access Initiative, Boston, Massachusetts, United States of America; 2 Sexual and Reproductive Health Team, Clinton Health Access Initiative, Accra, Ghana; 3 Independent Consultant, Yamba, New South Wales, Australia; 4 Family Health Division, Ghana Health Service, Accra, Ghana; PLOS: Public Library of Science, UNITED STATES OF AMERICA

## Abstract

Subcutaneous depot medroxyprogesterone acetate, or DMPA-SC, is an injectable contraceptive that can be administered by any trained person, including for self-injection. When scaling in-service healthcare worker training on DMPA-SC between 2019 and 2021, Ghana Health Service tasked a cohort of formally-trained healthcare workers to transfer their training ‘on-the-job’ to colleagues at their facilities, to save costs while improving training coverage. This cross-sectional mixed-methods implementation study explored the consistency of this approach in practice and how healthcare worker knowledge of DMPA-SC differed between healthcare workers receiving formal and on-the-job training. The study, conducted in 2021, included a structured survey of healthcare workers (N = 192) across trained facilities in four regions in Ghana, plus key informant interviews with regional resource team members (n = 8), facility in-charges (n = 8), and formally-trained or on-the-job trained healthcare workers (n = 16). Descriptive statistics, equality-of-proportions tests, and logistic regressions (adjusting for clustering by facility) were used to compare quantitative outcomes between formally-trained and on-the-job trained healthcare workers. Qualitative results were analysed thematically and triangulated with quantitative results. Only 62% of eligible healthcare workers surveyed reported receiving any on-the-job training on DMPA-SC from a formally-trained colleague. Where implemented, on-the-job training was reported to vary in length and depth, with on-the-job trained healthcare workers typically reporting fewer training components and lower satisfaction with training than formally-trained counterparts. Both cohorts scored comparably in injectables counselling role-plays and demonstrated comparable attitudes towards injectables. However, formally-trained healthcare workers scored better in overall correct knowledge of DMPA-SC (59.6% versus 26.4%, aOR: 4.68, 95%CI: 1.98-11.07, p < 0.01) and recall of all five critical self-injection steps (65.4% versus 37.9%, aOR 3.52, 95%CI: 1.53-8.10 p < 0.01). This study found that on-the-job training had been inconsistently implemented, seemingly leading to incomplete knowledge transfer on DMPA-SC between healthcare workers. The authors recommended standardising on-the-job training and increasing supportive supervision to strengthen the approach.

## Introduction

Intramuscular depot medroxyprogesterone (DMPA-IM) is the most popular contraceptive method in Ghana representing 38.4% of the method mix [1]. In 2017, a new injectable contraceptive product - subcutaneous depot medroxyprogesterone acetate (DMPA-SC) - was registered with the Food and Drug Authority (FDA) of Ghana. With a smaller needle and all-in-one Uniject system, DMPA-SC is simpler to administer than DMPA-IM and the FDA in Ghana approved DMPA-SC with a label including indication for client self-injection (SI) as well as for provider-administration (PA). DMPA-SC, including for SI, has been demonstrated as a feasible and acceptable method to clients and healthcare workers (HCWs) in a range of contexts, with the potential to increase continuation rates among injectable users and reduce the burden of repeat visits to facilities [2–9].

Following the registration of DMPA-SC in Ghana, the Ghana Health Service (GHS) collaborated with Population Council between 2017 and 2018 in the Ashanti and Volta regions to conduct a feasibility and acceptability study around a pilot of DMPA-SC, including for SI [10]. This included a comprehensive three-day in-service classroom-based training (50% theory, 50% practice) for the HCWs in the pilot. The study demonstrated high rates of feasibility and acceptability for DMPA-SC, including for the SI option, among HCWs and clients [10].

Following the dissemination of the pilot study results, GHS developed a national strategic introduction and scale-up plan for DMPA-SC (for both PA and SI) in 2019 [11]. Under Phase 1 of the plan, several strategic objectives were achieved between 2019 and 2020, including integration of DMPA-SC into the in-service HCW training curriculum. Formal in-service HCW trainings conducted between Q1 2019 and Q4 2020 trained 4,052 HCWs on DMPA-SC for both PA and SI, reaching 1,905 (19%) of 9,815 public and private facilities nationwide with at least one formally-trained (FT) HCW by the end of 2020. Despite resource mobilization efforts, the training roll out progress between Q1 2019 and Q4 2020 fell short of the national plan’s Phase 1 target to train 10,000 health workers across public and private sectors due to funding constraints. It has been estimated that when introducing a new contraceptive method, training HCWs is one of the largest cost drivers, often representing 50% or more of programme cost [12].

In a funding gap analysis conducted by Clinton Health Access Initiative (CHAI) on behalf of GHS in 2020, it was found the average cost to train one healthcare worker via formal training was around $79. At this cost, GHS faced a funding gap of $516,165 to meet their training targets for DMPA-SC, which created the need to consider alternative solutions to expand training coverage, while saving costs associated with formal training. The approach taken by GHS was to ask HCWs receiving formal training in 2020 to pass on their training content and new skills to other colleagues within their facilities via ‘on-the-job’ training. While peer-training has a limited evidence base in the literature, there is some evidence to support the idea that trainings at a HCW’s work site that include hands-on clinical components can be effective [13]. With few alternatives at the time, GHS decided to implement the on-the-job training (OJT) approach, leveraging their cohort of FT HCWs. Assuming each FT HCW was able to train one or two colleagues on-the-job at their facilities, the aim of this cascaded training approach was to double or triple the coverage of DMPA-SC trained HCWs, without adding substantial additional costs. However, prior to this study, it was unclear how consistently the cascaded OJT approach had been implemented in practice, nor how much of the FT HCWs’ knowledge of DMPA-SC had been retained and transferred to OJT-eligible colleagues.

Analysis conducted in early 2021, prior to this study, indicated that although 1,905 facilities were reportedly staffed with at least one DMPA-SC FT HCWs as of Q4 2020, the number of facilities reporting DMPA-SC clients (both PA and SI) into the national health information system (DHIMS) had never exceeded 900 facilities per month, at that point, and averaged only 645 facilities per month in Q1 2021. On average, only 16% of facilities providing any DMPA-SC each month were reporting provision of DMPA-SC for SI specifically. It was unclear at the time if this discrepancy reflected challenges with accurate data capture for DMPA-SC, poor retention of knowledge among FT providers, gaps in implementation of the OJT approach, and/or HCW attitudes towards the SI option.

GHS field visits across Ghana prior to this study had raised concerns that the OJT approach was being implemented inconsistently in practice, leading to poor quality of client counselling on DMPA-SC, particularly for the SI option. While the rate of clients preferring the SI option can vary across contexts depending when clients are asked [4,14], studies from other contexts have also shown that considerable time is often needed to train and reassure women about this new concept [5,7], which can place a burden on busy HCWs [15]. GHS hypothesised that HCWs receiving OJT may be even less inclined than their FT colleagues to see the value of SI and to invest this time in training women on SI.

In 2021, as GHS and partners started to review progress under Phase 1 of the national DMPA-SC roll out plan, they wanted to understand how consistently the directive to provide OJT had been followed, whether knowledge and skills transfer on DMPA-SC had taken place between FT and OJT-eligible HCWs, and whether knowledge, skills and attitudes towards DMPA-SC (including for SI) differed between HCWs receiving different training approaches.

### Research objectives

GHS’ aim with the OJT directive was to enhance training coverage for DMPA-SC by asking FT HCWs to share the content of their training with eligible colleagues, while saving costs. As part of a wider study looking at experiences of DMPA-SC provision and uptake in Ghana, this study component focused on the following objectives:

1. Explore if, when and how consistently OJT had been provided to OJT-eligible HCWs at sites with at least one FT HCW
2. Explore perspectives of RRT members, FT and OJT HCWs on the OJT approach, including length and depth of training received, satisfaction with training, and reasons why OJT had or had not been provided at sites with at least one FT HCW
3. Where feasible, compare post-training correct knowledge of DMPA-SC and the five SI critical steps among FT HCWs and OJT HCWs, to explore the extent of knowledge transfer via the OJT approach
4. Where feasible, compare HCW attitudes towards DMPA-SC for self-injection among FT and OJT HCWs, to understand if and how attitudes towards injectables, including for DMPA-SC SI, may differ between the two cohorts

## Materials and methods

### Implementation strategy

The formal training on DMPA-SC delivered in Ghana prior to the study (2019–2021) consisted of a classroom component, facilitated by Regional Resource Team (RRT) members, that was similar to that conducted during the original Population Council DMPA-SC pilot, but conducted in two days instead of three. Typically, only one HCW per facility was formally trained in this way - except in some higher-level facilities, where two or more HCWs may have been selected. GHS relied on District Directorates to advise on selected facilities from their district to be invited to participate in FT, while they in turn relied on facility in-charges to select one HCW to attend FT. These formal trainings were rolled out during a period of substantial health system disruption - both due to the COVID-19 pandemic in 2020 and a global supply disruption issue with DMPA-SC in 2021 – where resources were tight and governments were having to make the best of what they had available.

The formal classroom-based training curriculum was adapted from materials developed by the Access Collaborative and consisted of an overview of DMPA-SC, empathetic counselling and the HCW’s role in screening clients for DMPA-IM and DMPA-SC, storage and disposal of Unijects, and opportunities for role play to train clients on SI. During the two-day training, participants were provided with supplies, including a condom model for demonstration and training, a safety box, sample DMPA-SC Unijects, and materials to support implementation, such as observation checklists. With these materials, RRT members verbally encouraged the FT HCWs to share their new knowledge and skills with eligible colleagues at their facility through ‘on-the-job’ trainings (OJT), leveraging the same content and materials they had received via their formal trainings. However, no aspect of the formal training curriculum explicitly focused on building FT HCWs’ skills to deliver OJT, no additional materials or resources were shared with FT HCWs to deliver OJT beyond what they had received for their personal use, and no explicit guidance on the expected length, breadth or timeliness of OJT was shared with FT HCWs. Where funding allowed, RRTs in each region had a mandate to follow-up with facilities where a FT HCW was stationed to provide supportive supervision, including tailored support in delivering and strengthening OJT.

The ultimate aim of the OJT directive was for FT HCWs to pass on the knowledge and skills they had gained to any and all colleagues within their facility involved in contraceptive care, thus expanding the coverage of trained HCWs knowledgeable and skilled in provision of DMPA-SC, including for SI, and all while saving costs.

### Research design

This cross-sectional observational implementation study comprised a parallel convergent mixed-methods approach, drawing on:

1. Quantitative structured survey with HCWs (both FT and those eligible for OJT training – regardless of whether they had received OJT) at sampled public and private facilities
2. Qualitative key informant interviews (KIIs) with stakeholders involved in provision of in-service training, namely RRTs; facility in-charges at sites with at least one FT HCW; and a sub-sample of family planning (FP) HCWs (both FT and OJT)

### Research outcomes

The primary outcomes of interest were post-training correct knowledge of DMPA-SC (defined as >85% correct answers, adapting the post-training knowledge tests used during FT) and accurate unprompted recall of the five critical steps for SI (as defined by GHS’ in-service formal training manuals). As FT HCWs were tasked to use their own training content and materials to provide OJT to colleagues, in theory OJT HCWs being assessed against the same post-training knowledge tests as FT HCWs should demonstrate whether complete knowledge and skills transfer had occurred via OJT.

Other outcomes of interest included: consistency of provision of OJT to eligible colleagues by FT HCWs, including reported timing and components of training; RRTs’ and HCWs’ perspectives on FT and OJT training received, including satisfaction with training and reasons for providing or not providing or receiving OJT; and HCWs’ attitudes towards injectables, including DMPA-SC for SI. While cost-effectiveness of the OJT approach was hypothesised by GHS, this was beyond the scope of the research to assess.

### Study sites and samples

At the time of designing the study, some of Ghana’s 16 regions had slightly higher coverage of ‘trained sites’ (i.e., higher percentage of all contraceptive-providing sites with at least one DMPA-SC FT HCW) than others, ranging from under 5% to over 25%. Four regions for the study were purposively sampled based on being the region(s) in each Belt with the highest coverage of ‘trained sites’. This approach was taken to ensure inclusion of facilities (and therefore HCWs) with sufficient exposure to the DMPA-SC in-service training to provide valuable insights. Two regions were sampled from the Southern Belt as it had the highest concentration of trained HCWs in the country. The sampled regions were:

- Southern Belt: Central and Eastern regions (26% and 17% coverage of trained sites at the time of the study, respectively)
- Middle Belt: Ashanti region (17% coverage)
- Northern Belt: Upper East region (9% coverage)

Within the four selected regions, districts with at least 10% training coverage (measured as the number of sites with at least one FT HCW divided by total number of sites providing contraceptive services) were included in the sampling frame (n = 101 eligible districts out of a possible 113). Minimum required sample size estimates to detect a difference of 20 percentage points or more in proportion of correct DMPA-SC knowledge between FT and OJT-eligible populations were generated assuming an average cluster size of three HCWs per site (where one HCW was assumed to be FT and two were assumed to be OJT-eligible) and an intraclass correlation of 0.5, with alpha set to 0.05 and beta set to 0.20 (80% power). Based on this, it was estimated that a minimum of 62 clusters (facilities) was needed across 16 districts, which would provide a minimum of 62 FT HCWs and 124 OJT-eligible HCWs (total minimum N = 186). For the purposes of easy division for probability proportional to size (PPS) sampling, this was increased slightly to 64 facilities across 16 districts.

The 16 districts in the four regions were sampled using PPS, such that districts with a larger population of ‘trained sites’ were more likely to be sampled. Then, within each of the 16 districts sampled, an even number of facilities (four per district) with at least one FT HCW were randomly sampled. Within each site, all FT HCWs and OJT-eligible HCWs were eligible and invited to be surveyed, including those who were OJT-eligible but untrained/self-trained, as it was not possible to estimate *a priori* what percentage of the OJT-eligible population would have actually received OJT. It was known that the sample would lose power to detect the desired percentage point difference in knowledge outcomes if the number of HCWs actually receiving OJT was substantially lower than the total OJT-eligible population, but budget limitations meant it was not possible to further inflate the sample to adjust for this possibility.

The sampling approach for the HCW survey did not aim to be nationally representative*.* Instead, it aimed to be representative only of HCWs at ‘trained sites’ (i.e., with at least one FT HCW) in the four purposively sampled regions. No stratification by region was conducted. Challenges around sampling are outlined in the Limitations section.

The qualitative sample was recruited from a sub-sample of the same 64 sites and spread evenly across the four regions to ensure perspectives from each region. The final qualitative sample size was based on theoretical saturation, which refers to the point at which no new concepts emerge from qualitative data drawn from a sample that is diverse in pertinent experiences and characteristics [16]. In order to assess whether theoretical saturation had been achieved, transcription was conducted in an iterative way throughout the data collection process and initial transcripts reviewed by the coding team. Table 1 outlines the final quantitative and qualitative sample sizes.

**Table 1:** Sample size by population and data collection method.

Study population	Data collection method	Sample
FP HCWs working at sites with at least one FT HCW (i.e., ‘trained sites’)	Structured HCW survey	192 (52 FT, 140 OJT-eligible, of whom 87 had actually received some OJT, leaving 53 untrained/ self-trained)
Regional resource team (RRT) members	Key informant interviews	8 (2 per region)
Facility-in charges at trained sites	Key informant interviews	8 (2 per region, 3 OJT trained, 5 FT trained)
FT or OJT trained HCWs	Key informant interviews	16 (4 per region, 7 OJT and 9 FT)

### Data collection

Quantitative data collection tools for the HCW survey were developed to align with content of the GHS’ in-service training curriculum on DMPA-SC and the post-training knowledge assessment used during FT. The survey included questions on experience of FT or OJT training (if received); a counselling role-play where the data collector played the role of a ‘client’ interested in injectables and scored the HCWs according to set criteria from GHS counselling guidelines; a client-training role-play where the data collector played the role of a ‘client’ interested in learning to SI and scored the HCWs on their recall of the five critical SI steps as defined in GHS training manuals (1: select appropriate injection site and clean it if needed; 2: Mix the solution by shaking the device for about 30 seconds; 3: Activate the device by closing the gap between the needle cap and port; 4: Gently pinch the skin at the injection site to form a ‘tent’; 5: Insert needle completely so that port touches skin and press the reservoir slowly to inject for about 5–7 seconds); and finally Likert scale questions assessing HCW attitudes towards injectables. Qualitative KII discussion guides were semi-structured and developed based on a theoretical framework mapping out a HCW behaviour change journey towards integration of DMPA-SC, including for SI, into routine care. This framework was developed from a rapid literature review conducted prior to the study and is outlined in S1 Table.

Qualitative and quantitative data collectors participated in a three-day training workshop, including training on how to assess injectables counselling and SI training role plays against the assessment criteria, as well as an opportunity to pre-test and revise the study tools. Data collectors were also trained to translate the questionnaires and discussion guides into Akan or Frafra, as needed. Subsequently, participants were recruited and surveyed and/or interviewed over a two-week period (1^st^ to 12^th^ November) in 2021. In practice, several site substitutions had to be made due to incorrect information from the sampling frame about the presence of a FT HCW. In addition, the study team encountered sites where an FT HCW had been in post (and may or may not have trained colleagues OJT) but had either moved facilities or was unavailable during the site visit (for example, on leave, attending another training, or not on shift), meaning they were unable to survey the FT HCW on the day of the visit – see the Limitations section of this paper for more details.

Response rates were high, with very few cases of HCW refusal to participate. Quantitative data was collected through tablets into open-source software (Kobo Collect), while qualitative data was audio-recorded and transcribed verbatim in the language of interview (mostly English, although some were conducted in Akan or Frafra before being translated into English for analysis).

### Data analysis

Quantitative data was exported from Kobo Collect, cleaned and analysed as descriptive statistics initially in SPSS by one member of the analysis team (ST). Differences in binarised versions of the primary outcomes between the FT and OJT sub-groups in the HCW survey were explored through equality-of-proportion tests (adjusted for clustering by facility) and then further investigated through mixed-effects logistic regression analysis (again controlling for clustering by facility) in STATA-17 by another member of the team (CPE). Sensitivity analyses of key outcomes were conducted where years of clinical experience was also controlled for in the logistic regression models, however this did not appear to be a significant confounder in most cases. Missing data was minimal in the quantitative survey dataset, limited to some of the variables around training length and some attitudes statements.

Three members of the study team (CPE, MS and ST) coded 25% of the qualitative transcripts for each of the three KII populations deductively at first against a draft set of codes developed based on the HCW theoretical framework in S1 Table. These independent code applications were then compared to check for consistency and additional inductive codes added to create a comprehensive codebook. Once consistency in code application was achieved between the coding team, the final codebook was then applied to all remaining transcripts by the same team of coders. Dedoose software was used for data coding, organization and retrieval. All quantitative findings were then triangulated with qualitative findings by four members of the team (CPE, ST, MS, MBE) for contextualization, in line with a parallel convergent mixed-methods approach.

Validation workshops with key stakeholders in the study (RRT members, public health nurses, selected facility in-charges, and selected FT and OJT HCWs) were conducted in two study regions (Ashanti and Eastern) in June 2022 to share the draft findings, check interpretation, and help with generating recommendations.

### Ethics statement

This study was conducted in line with the principles outlined in the Declaration of Helsinki. The study team worked closely with the study regions, districts and facilities through the office of the GHS Director General to secure appropriate approvals. The study protocol and tools were approved by GHS Ethics Review Committee in Accra, Ghana, a board that is also registered as an International Institutional Review Board with Federal Wide Assurance (IRB: IRB00009260, FWA00020025), study protocol approval number GHS-ERC: 019/08/21. All participants provided written informed consent to participate in the survey and KIIs in their preferred language.

## Results

### Participant characteristics

In practice, an average of three contraceptive-providing HCWs were surveyed per facility, though this ranged from one to seven HCWs per site, depending on the size of the site and HCW availability on the day of the visit. In practice, a mean of 0.8 FT HCWs per site were surveyed (total n = 52, median per site = 1, range per site 0–2). This represents 81% of total eligible FT providers at the sites (52 out of 64), reflecting that not all FT providers were available to survey on the day of the visit. A mean of 2.2 OJT-eligible HCWs were surveyed per site (total n = 140, median per site = 2, range per site 0–6). Of the OJT-eligible population, a mean of 1.4 per site had actually received some OJT (total n = 87, median per site = 1, range 0–4), and a mean of 0.8 per site had received no training at all from their FT colleague (total n = 53, median per site = 0, range 0–4). It is unknown how many OJT-eligible HCWs were unavailable on the day of the visit. The mean number of FT HCWs surveyed per district was 3.3 (median per district = 3.5, range 1–4); the mean number of HCWs actually receiving OJT per district was 5.4 (median per district = 5.0, range 3–9); and the mean number of HCWs eligible for OJT but receiving no training per district was 3.3 (median per district = 3.0, range 0–9).

The structured HCW survey included a cadre mix of FP HCWs and facility in-charges (n = 192) from the 64 sampled facilities - the majority (69.3%) were community health nurses (CHNs), while 16.7% were nurses, 10.4% were midwives and 3.6% had other cadres (for example, field technicians or health assistants). The 64 facilities represented 58 public and 6 private facilities.

Facility-in charges and HCWs (n = 24) participating in the KIIs included a mixture of CHNs, midwives, and nurses. They reported working at a range of public and private sites - mainly community-based health planning and service (CHPS) facilities or health centres and hospitals in a few cases.

All sampled RRTs (n = 8) were involved in the facilitation of the DMPA-SC in-service formal training in their region; some had additionally been involved in routine supportive supervision visits since the trainings.

### Implementation of the OJT approach in practice

In the survey, nearly three quarters (72.4%) of HCWs had received some DMPA-SC training (27.1% FT plus a further 45.3% OJT) while the remaining 27.6% had not been trained yet, despite working at a site with at least one FT colleague (Table 2). One HCW surveyed did not receive any training but was self-taught via the internet - this HCW was grouped with the untrained group for further analysis. This meant that of all OJT-eligible HCWs (n = 140), only 62.1% (n = 87) had received any OJT in practice - despite most FT HCWs having received their FT between seven and twelve months prior to the survey (Table 2).

**Table 2:** Survey responses about training on DMPA-SC.

Percentage of each trained sub-group reporting length of training				
Training length among trained HCWs	% untrained/ self-trained HCWs (n = 53)	% OJT HCWs (n = 87)	% FT HCWs (n = 52)	% total (N = 139)
1-day session	N/A	70.1	34.6	56.8
Session of 2 consecutive days	N/A	12.6	42.3	23.7
Session of 3 consecutive days	N/A	3.4	5.8	4.3
Conducted in multiple sessions spread out over a length of time	N/A	5.8	1.9	4.3
Missing response	N/A	8.1	15.4	10.8
Total	N/A	100.0	100.0	100.0
**Percentage of each sub-group reporting timing of training**				
**Time since DMPA-SC training**	**% untrained/ self-trained HCWs** **(n = 53)**	**% OJT HCWs** **(n = 87)**	**% FT HCWs** **(n = 52)**	**% total (N = 192)**
Training is planned or ongoing	100.0	0.0	0.0	27.6
Within the last month	0.00	40.2	17.3	22.9
1-6 months ago	0.00	29.9	11.5	16.7
7-12 months ago	0.00	28.7	71.2	32.3
More than 12 months ago	0.00	1.2	0.0	0.5
Total	100.0	100.0	100.0	100.0
**Percentage of untrained/self-trained and FT HCWs reporting reasons for not receiving or not giving OJT**				
**Reasons given for not receiving OJT** ^**a**^	**% untrained/ self-trained HCWs** **(n = 53)**	**Reasons given for not providing OJT** ^**a**^	**% FT HCWs who trained none or only some eligible colleagues (n = 34 out of 52)**	
Training is planned but has not happened yet	30.8	Training is planned but has not happened yet	28.6	
Lack of time/ FT HCW too busy to train	26.9	Lack of time/ FT HCW too busy to train	3.6	
FT HCW unavailable for training	13.5	OJT-eligible HCW unavailable for training	35.7	
FT HCW uninterested in providing training	1.9	OJT-eligible HCW uninterested in training	0.0	
In-charge unsupportive of training	0.0	In-charge unsupportive of training	0.0	
Insufficient tools to train	17.3	Insufficient tools to train	25.0	
FT HCW lack of confidence in DMPA-SC	0.0	FT HCW lack of confidence in DMPA-SC	0.0	
Insufficient client demand for DMPA-SC to practice	3.9	Insufficient client demand for DMPA-SC to practice	7.1	
Not FT HCW’s job to train colleagues	0.0	Not FT HCW’s job to train colleagues	0.0	
FT HCW prioritized some colleagues for training over others	3.9	FT HCW prioritized some colleagues for training over others	0.0	
OJT-eligible HCW started at the facility too recently, missed OJT	11.5	No other FP HCWs at facility for FT HCW to train	17.9	
OJT-eligible HCW unaware there was an FT HCW	9.6	Conducted OJT but those colleagues moved on	2.6	
No stock of DMPA-SC to aid training	3.9	No stock of DMPA-SC to aid training	2.6	
Don’t know why	1.9	Don’t know why	0.0	

^a^multiple choice allowed - columns will not add up to 100%.

FT HCWs had on average the highest number of years of experience (6.7), compared to HCWs receiving OJT (mean = 5.2 years) and OJT-eligible but untrained/ self-trained HCWs (mean = 4.1 years). Most FT HCWs recalled receiving their two-day training, however around a third recalled their training as only one day – likely due to recall bias or perhaps having only attended part of the two-day formal training. Both FT HCWs and untrained/self-trained HCWs in the survey were asked to indicate the main reasons for not yet providing or receiving OJT - with most agreeing that colleague unavailability and insufficient tools were the main reasons (Table 2).

In KIIs, FT HCWs’ experience of implementing the OJT varied, with a few FT HCWs reporting actively running regular (for example, twice per year) trainings for their colleagues, while the majority reported holding only one initial post-training ‘briefing’ for their colleagues. For their part, most OJT HCWs in the survey reported receiving a one-day training (Table 2) which they clarified in KIIs was typically just a single short session or ‘briefing’ on DMPA-SC, with the chance to ask questions and perhaps a demonstration with one Uniject, where samples were available.

**Fig 1:**
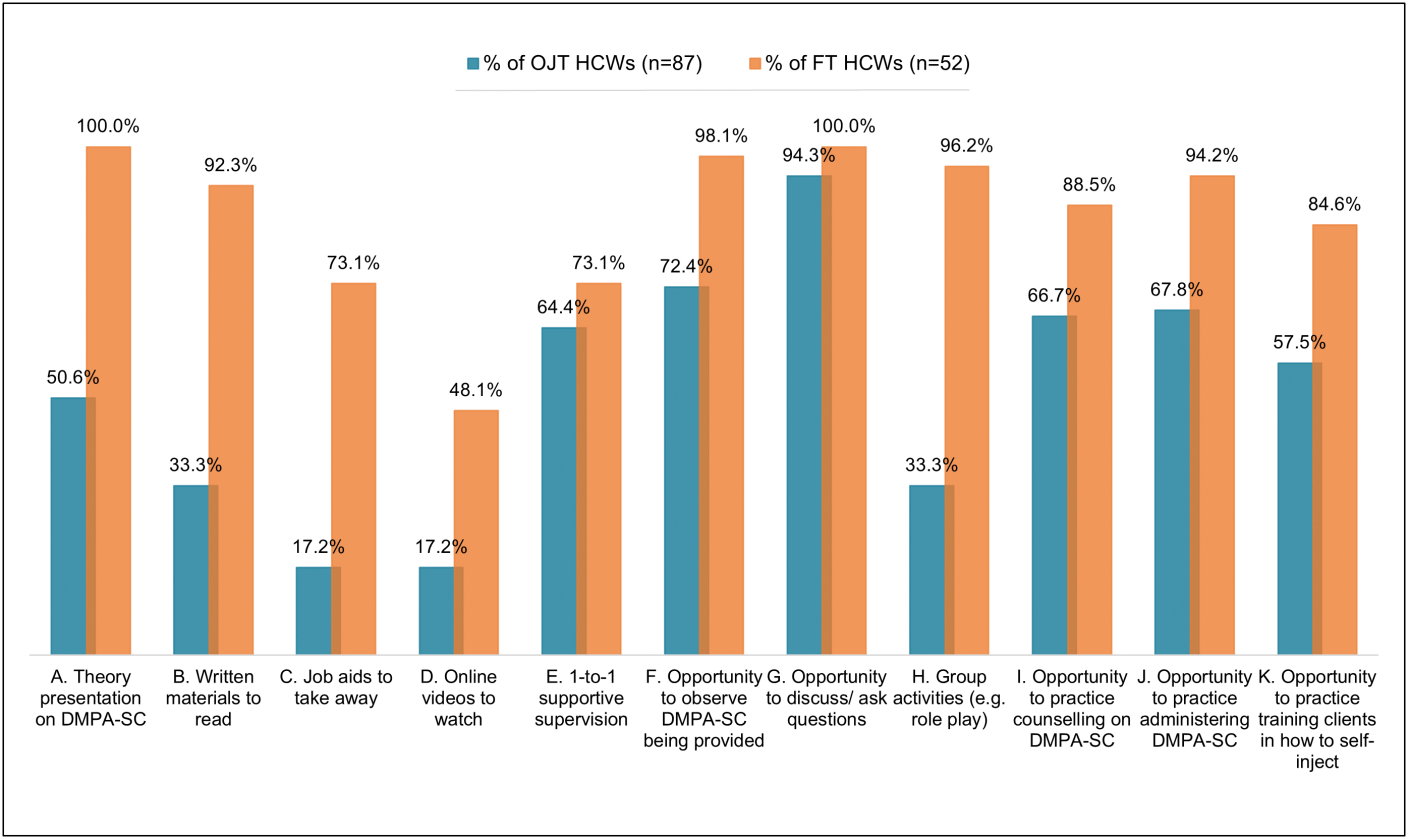
Percentage of respondents reporting specific training components, by training type.

FT HCWs answering the survey were more likely to report receiving a wide range of elements of the training compared to OJT HCWs (Fig 1). All differences in Fig 1 were statistically significant (p < 0.01) in equality-of-proportion tests, except for ‘E) one-to-one supportive supervision’ (p = 0.31) and ‘G) the opportunity to discuss/ask questions’ (p = 0.08).

Just over half (56.8%) of HCWs/in-charges said that their facility was providing DMPA-SC at the time of the survey, while 30.7% said it was not, and 12.5% said they did not know. In the survey, 40.4% of HCWs who were aware of DMPA-SC being provided at their facility (n = 109) said they had faced challenges with injectables stock-outs in the last three months (68.2% of these said the issue was with DMPA-SC, while 15.9% said DMPA-IM and 15.9% said both injectable products). In the KIIs, some HCWs reported not receiving any DMPA-SC commodities at all since being trained. Others only received limited commodities that were meant to be used for demonstration during OJT, while a few said initial stocks for service provision ran out after a few months. Aside from disrupting provision of DMPA-SC, some HCWs also talked about stockouts having disruptive effects on FT HCWs’ ability to train their colleagues OJT:

*“I have been able to talk to my colleagues [provide OJT training] … I have explained things to them that this is how it [Uniject] is, this is how it’s been used and the side effects and other things, but they have not yet seen the commodity nor practiced with it.” -* ***FT HCW, Ashanti***

### Healthcare workers’ satisfaction with training received

In the KIIs, all RRTs interviewed said that by the end of the DMPA-SC trainings they were confident the FT HCWs had grasped the method. Most RRTs and FT HCWs in the KIIs felt that the two-day length of the in-service FT was sufficient, because the participants were already experienced contraceptive-providing HCWs, while a minority of both groups felt that the trainings should be extended by another day to ensure sufficient time for practice.

**Fig 2:**
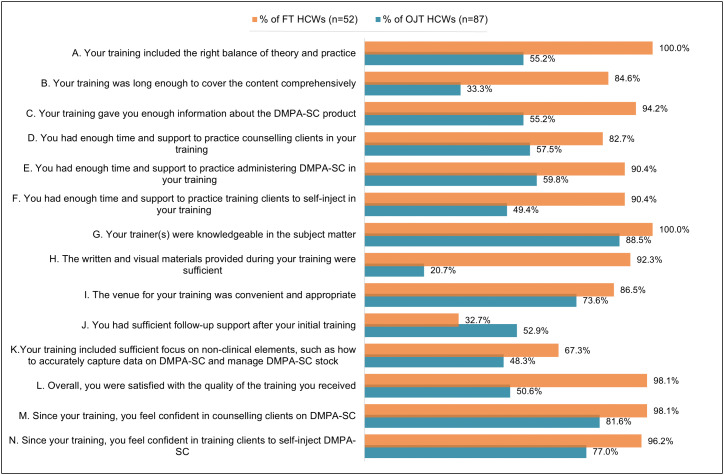
Percentage respondents agreeing/strongly agreeing to statements on quality of training received, by training type.

Over two thirds (68.4%) of trained HCWs (FT and OJT, n = 139) in the survey reported overall satisfaction with their training, however this differed significantly between FT and OJT HCWs (98.1% versus 50.6% respectively, p < 0.01). With logistic regression, FT HCWs had nearly 90 times the odds of having overall satisfaction with their training than OJT providers (aOR 89.6, 95% CI: 7.73-1039.07, p < 0.01). In fact, OJT HCWs were statistically significantly less satisfied with almost every element of their training (p < 0.05) except J) post-training follow up support (where they were slightly more satisfied, p = 0.025) and I) venue (where the difference did not emerge as significant, p = 0.096) (Fig 2).

In KIIs, most FT HCWs were positive about the content of their training - they particularly appreciated the visual/written aids they were given, seeing the Unijects, and the opportunity for discussion and role plays with colleagues.

*“…the training materials were good, they showed us how the Sayana Press [DMPA-SC] is used, they even opened it and we did practical on it and the trainers were good.” –* ***FT HCW, Upper East***

For their part, while seemingly hesitant to criticise their FT colleagues, several OJT HCWs in KIIs indicated that the insufficient level of the information received and lack of stock (in some cases) to facilitate demonstrations and practice opportunities meant their DMPA-SC training was insufficient.

*“… when they [FT colleagues] came to train us, we used one [Uniject] for the training for example, but we didn’t inject on anybody so as to see how it is opened and how to even pierce [activate] it…” -* ***OJT HCW, Eastern***

### Healthcare workers’ post-training attitudes, knowledge and skills on provision of DMPA-SC

#### Healthcare workers’ attitudes towards injectables post-training.

**Fig 3:**
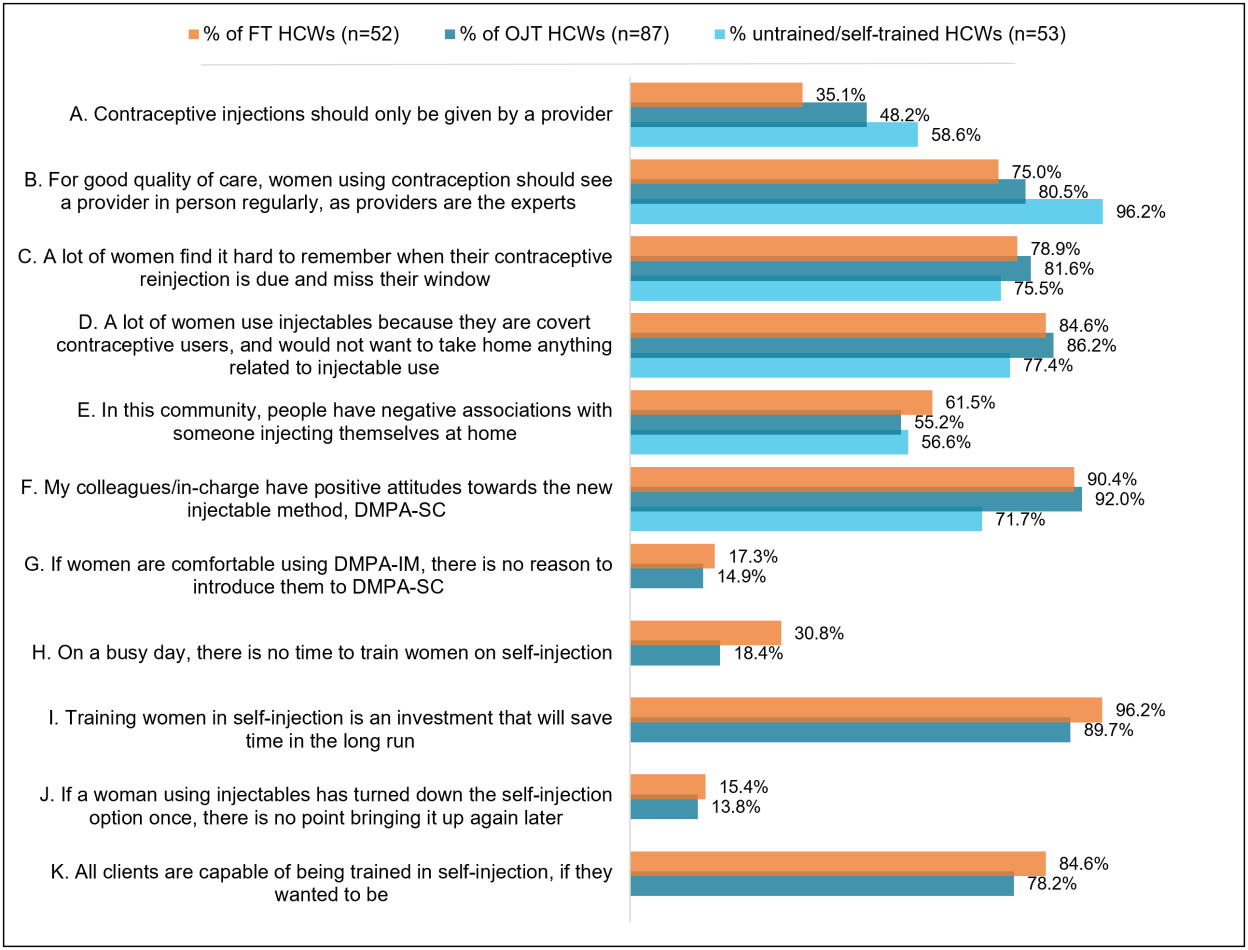
Percentage of respondents who agree/ strongly agree with attitudes statements, by training type.

The HCW survey included a series of attitudinal statements about injectable contraceptives and HCWs were asked if they agreed or disagreed with the statements. While untrained providers showed attitudes slightly less supportive of SI in particular, importantly, there were no significant differences between the percentages of FT and OJT HCWs agreeing or strongly agreeing with the statements, when tested with equality-of-proportion tests (all p > 0.05) (Fig 3).

#### Post-training knowledge retention on DMPA-SC.

In the survey, HCWs with any training (FT or OJT) showed better knowledge of DMPA-SC than the untrained/self-trained group - who unsurprisingly tended to respond that they did not know the answer in most cases (Table 3). When omitting the untrained/self-trained group, FT HCWs were statistically significantly more likely to score >85% correct answers (6 or 7 out of 7) on the DMPA-SC knowledge test than their OJT counterparts (59.6% FT versus 26.4% OJT, p < 0.01). With logistic regression, this meant FT HCWs had 4.7 times the odds of scoring >85% correct answers compared to OJT HCWs (aOR: 4.68, 95%CI: 1.98-11.07, p < 0.01).

**Table 3:** Survey results for DMPA-SC knowledge assessment.

DMPA-SC knowledge statements (n = 192)	Response (correct response in green)	% of untrained/ self-trained HCWs (n = 53)	% of OJT HCWs (n = 87)	% of FT HCWs (n = 52)
**a. DMPA-SC (Sayana Press) does not need to be activated before use**	TRUE	9.4%	19.5%	19.2%
	FALSE	11.3%	64.4%	76.9%
	I don’t know	79.3%	16.1%	3.9%
**b. DMPA-SC (Sayana Press) contains a lower dosage of active hormone compared to DMPA-IM**	TRUE	11.3%	35.6%	59.6%
	FALSE	15.1%	35.6%	32.7%
	I don’t know	73.6%	28.7%	7.7%
**c. Used units of DMPA-SC can be disposed of in latrines**	TRUE	9.4%	33.3%	13.5%
	FALSE	35.9%	58.6%	84.6%
	I don’t know	54.7%	8.1%	1.9%
**d. DMPA-SC differs from DMPA-IM because it can be injected into the skin, rather than the muscle**	TRUE	22.6%	63.2%	69.2%
	FALSE	9.4%	28.7%	28.9%
	I don’t know	67.9%	8.1%	1.9%
**e. DMPA-SC should not be used by women who are breastfeeding**	TRUE	7.7%	6.9%	9.8%
	FALSE	36.5%	82.8%	86.3%
	I don’t know	55.8%	10.3%	3.9%
**f. Clients who received DMPA-IM three months ago cannot switch to DMPA-SC during their reinjection window**	TRUE	3.8%	11.5%	9.6%
	FALSE	34.0%	82.8%	90.4%
	I don’t know	62.3%	5.8%	0.0%
**g. DMPA-SC protects against pregnancy for 1 month only**	TRUE	0.0%	2.3%	3.9%
	FALSE	43.4%	95.4%	92.3%
	I don’t know	56.6%	2.3%	3.9%
**Overall knowledge scores**	No knowledge	47.2%	0.0%	0.0%
	0-5 correct answers out of 7	43.4%	73.6%	40.4%
	6-7 correct answers out of 7	9.4%	26.4%	59.6%

#### Assessment of role-played injectables counselling.

In the survey, all HCWs were asked to role play a counselling session with a ‘client’ (the data collector) who was only interested in injectables. They were asked to use all the messages and props they would typically use with a real client. Data collectors were trained to score the HCWs’ counselling approach against a set of criteria (drawn from the national DMPA-SC training curriculum). Generally both FT and OJT HCWs scored better than untrained/self-trained HCWs – especially in terms of mentioning the SI option and offering to train the client in SI or refer them for SI training. While some minor differences emerged in the responses between the FT and OJT groups on injectables counselling, none were large enough to be detected as statistically significant in equality-of-proportion tests (all p > 0.05, Table 4).

**Table 4:** Survey results for assessment of injectable counselling and SI training role plays.

Data collector assessment of injectable counselling role play (N = 192)	Assessment outcome	% untrained/self-trained HCWs (n = 53)	% OJT HCWs (n = 87)	% FT HCWs (n = 52)
**HCW asked appropriate questions about fertility intentions and lifestyle**	Yes	43.4%	59.8%	61.5%
	Partially	18.9%	21.8%	19.2%
	No	37.7%	18.4%	19.2%
**HCW presented all injectable options equally and did not try to convince use of one method over another**	Yes	45.3%	78.2%	88.5%
	Partially	28.3%	13.8%	1.9%
	No	26.4%	8.1%	9.6%
**HCW explained differences between the injectable options clearly**	Yes	30.2%	65.5%	80.8%
	Partially	41.5%	26.4%	13.5%
	No	28.3%	8.1%	5.8%
**HCW clearly explained the pros and cons of each injectable option**	Yes	47.2%	63.2%	65.4%
	Partially	22.6%	20.7%	21.2%
	No	30.2%	16.1%	13.5%
**HCW mentioned all three injectable options (without prompting)**	Yes, all three	22.6%	64.4%	73.1%
	Mentioned one or both PA options but not SI	49.1%	27.6%	25.0%
	Did not specify that there are difference types of injectable	28.3%	8.1%	1.9%
**After ‘client’ revealed interest in SI, HCW offered to take time to train ‘client’ in SI *OR* refer ‘client’ to a colleague for SI training**	Yes, offered to train ‘client’ in SI	13.2%	82.8%	84.6%
	Yes, offered to refer ‘client’ for SI training	30.2%	6.9%	5.8%
	No, did not offer to train nor refer ‘client’ for SI training	56.6%	10.3%	9.6%
**Data collector assessment of self-injection training role play (n = 139)**	**Assessment outcome**	**% untrained/self-trained HCWs (n = 53)**	**% OJT HCWs (n = 87)**	**% FT HCWs (n = 52)**
**HCW mentioned all 5 critical SI steps** ^**a**^	At least 1 critical step missed	N/A	62.1%	34.6%
	All 5 critical steps mentioned	N/A	37.9%	65.4%
**HCW reassured and build ‘client’ confidence in being able to self-inject**	Yes	N/A	56.3%	71.2%
	Partially	N/A	31.0%	25.0%
	No	N/A	12.6%	3.8%
**HCW used client SI instruction guidelines**	Yes	N/A	37.9%	44.2%
	Partially	N/A	10.3%	19.2%
	No	N/A	51.7%	36.5%
**HCW used example reinjection calendar**	Yes	N/A	31.0%	42.3%
	Partially	N/A	18.4%	9.6%
	No	N/A	50.6%	48.1%
**HCW used example disposal container**	Yes	N/A	37.9%	65.4%
	Partially	N/A	17.2%	7.7%
	No	N/A	44.8%	26.9%
**HCW used models for SI practice (e.g., condom filled with salt)**	Yes	N/A	12.6%	34.6%
	Partially	N/A	9.2%	7.7%
	No	N/A	78.2%	57.7%
**HCW used example Uniject**	Yes	N/A	60.9%	69.2%
	Partially	N/A	12.6%	7.7%
	No	N/A	26.4%	23.1%

^a^Critical step 1: select appropriate injection site/ clean if needed; Critical step 2: Mix solution by shaking device for about 30 seconds; Critical step 3: Activate device by closing gap between needle cap and port; Critical step 4: Gently pinch skin at injection site to form a ‘tent’; Critical step 5: Insert needle completely so that port touches skin and press the reservoir slowly for about 5–7 seconds.

When probed in KIIs about whether they emphasized or omitted the SI option for particular types of clients, several HCWs did admit to using their own observations and judgments about which clients might be most interested in, or capable of, self-injecting. For some of these HCWs, this was more about assessing which clients might be sufficiently motivated by the benefits of SI to overcome fears (for example, those who lived far away, or travelled often). However, among a minority of HCWs, this judgment call seemed to reflect HCWs’ lack of trust that women with low levels of literacy would be able to SI.

*“…we [HCWs] had some concerns because majority of our clients are also not literate so and most of the time some may even miss their [reinjection] dates … That was our major concern… How to even teach them to understand the procedure [self-injection]… Because you take the person through the training, and you say ‘oh, do it, let me see’ and then the person is all over the place.” -* ***FT facility in-charge, Eastern***

#### Assessment of role-played self-injection client training.

After role-playing an injectable counselling session, HCWs in the survey were then asked to assume the ‘client’ (the enumerator) was interested in the SI option and proceed to SI training. Again, enumerators were trained to assess the HCWs’ approaches against set criteria, drawn from the national DMPA-SC training curriculum. Overall, 48.2% of trained HCWs (FT and OJT) mentioned all five critical SI steps, unprompted, during the role play. However, this was statistically significantly different between FT and OJT HCWs (65.4% and 37.9% mentioning all five critical steps respectively, p < 0.01). With logistic regression, this meant that FT HCWs had 3.5 times the odds of recalling all five critical SI steps correctly, compared to OJT HCWs (aOR: 3.52, 95% CI: 1.53-8.10, p < 0.01). In addition, OJT HCWs in the survey appeared to be significantly less likely to use an example disposal container and demonstration models than FT HCWs (both differences p < 0.01 in equality-of-proportions tests). There were no other statistically significant differences detected between FT and OJT HCWs in the SI training indicators from Table 4.

In KIIs, most HCWs interviewed (both FT and OJT) reported struggling with introducing the SI concept to women and reassuring them enough to SI. A small number of HCWs (FT and OJT) reported improvising techniques to support women’s confidence with SI, such as showing women how small the Uniject is, using visual aids or gestures, conducting repeated demonstrations, and leveraging the testimonies of experienced SI clients. While most HCWs in KIIs were positive about the idea of visual aids to help clients understand and recall the SI steps, many did not appear to have access to any visual aids - if they did, these were mainly one or two items left over from their own formal trainings.

Only a few (mostly FT) HCWs mentioned that they regularly include SI demonstrations and/or practice opportunities for women, such as using condoms filled with salt or improvised materials (e.g., oranges), in their training approach. By contrast, the majority of HCWs in KIIs either did not mention conducting demonstrations/practice sessions on inanimate materials or said they preferred not to conduct them, seemingly worrying about ‘wasting’ DMPA-SC stock on demonstrations and practice sessions. Instead, some HCWs talked about demonstrating how Unijects worked through PA on a real client and/or asking women to directly SI under observation without prior practice on inanimate objects.

### Reflections on the OJT approach and the role of supportive supervision

The majority of RRTs felt that the post-training supportive supervision for the DMPA-SC in-service training roll out had been inadequate and a few attributed this to lack of funding. Two RRT members noted that this follow-up aspect was particularly important in the OJT approach, to quality assure the level of OJT provided by FT HCWs and correct any misunderstandings.

*“… it’s not necessary trying to bring all of them [HCWs] to a place to train but you train few and there should be a follow up …, you go for supervision … [of] those who were trained, you can re-access them and you get the chance also to train the new ones and you also find out that when they came [back], were they able to train others [OJT], so that you know their weakness and then you build it up, they should be [subjected to] frequent supervision...” –* ***RRT Upper East***

On their side, FT and OJT HCWs had mixed experiences of receiving follow-up supportive supervision (sometimes described instead as mentorship or monitoring visits) after their trainings. Around half reported receiving supportive supervision visits from a variety of sources - some from GHS, others from non-governmental organisations. The other half of FT and OJT HCWs said they had not received any follow-up support, except some ad hoc advice from peers or facility in-charges.

Even among those who received a post-training visit, they often expressed the need for more frequent visits. In particular, those requesting more supportive supervision wanted more support with how to introduce DMPA-SC to women in a way that reassures them about the SI option.

*“… the supervisors … they have to come and visit us, to visit the communities to talk about the commodity… The supervisors’ have to come along with us when we go for outreach like this… so they can also talk to them [clients] for us.” -* ***OJT HCW, Central***

All participants (RRTs, facility in-charges and HCWs) were asked to reflect on the effectiveness and functionality of the OJT approach. The majority of RRT members were positive about the potential cost-savings of the approach, but some worried about the quality of information reaching OJT HCWs. The majority of FT HCWs were also worried about the quality of OJT they were passing on to their colleagues.

*“I mean [in terms of] getting to train everybody, it [OJT approach] will save time and money and then … the negative aspect of it will be that probably the person who was present at the workshop may not be able to … relay the information and be able to train the others quite properly. That may be a disadvantage.”* ***– FT facility in-charge, Eastern***

Most OJT HCWs also expressed concern that the information they had received was incomplete.

*“I think we were trained on the job [with] this thing [DMPA-SC] … Maybe what our [FT] in-charge taught us was… she can omit something and so when the opportunity comes, [maybe] you can train some of the [other] staffs…”* ***- OJT HCW, Ashanti***

By contrast, a few FT HCWs and one OJT HCW described examples of the OJT approach working well in their facility. These examples seemed to be reflective of the minority of cases where an experienced trainer was selected as the FT HCW, and where these FT HCWs were running regular (for example, twice per year) and practice-based OJT:

*“So it’s like twice a year, we go through that … And so since they came [back from FT], they’ve been practicing and … they [FT HCWs] are also training their… colleagues. So it’s like they are flowing together. We haven’t had any challenge...” -* ***FT facility in-charge, Central***

In fact, one FT HCW said the OJT approach had the benefit of there being real clients to work with, and continuous practice opportunities, as long as there was DMPA-SC stock available.

## Discussion

This study explored the implementation of a cascaded OJT approach aiming to increase training coverage for DMPA-SC in the public sector across four regions in Ghana, while saving training costs.

Most FT HCWs (but not all) appear to have provided some form of OJT to their eligible colleagues upon their return to the facility, however this varied in timeliness, breadth and quality. Where OJT was not being provided at all, this mainly appeared to be due to lack of stock/tools to train, HCW attrition or leave, and busy HCW schedules. Where OJT was being provided, in most cases it seemed to consist of a short, one-off post-training debrief session by the FT HCW, often without visual or written materials and sometimes also without DMPA-SC stock. It’s likely that the quality of OJT was further affected by delays in provision of OJT by FT HCWs – with many FT HCWs seemingly waiting several months before passing on their knowledge.

Stock issues were a major challenge at the time of the study that had knock-on effects on all areas of integration of DMPA-SC, including the prioritisation of OJT and HCW confidence in the product. In 2020, a global disruption to the manufacturing process of the sole supplier led to limited commodity availability, which constrained the scale-up and broader distribution of DMPA-SC. A manufacturing backlog, high demand worldwide, and product registration requirements in Ghana prevented the timely shipment of additional orders of DMPA-SC in the latter half of 2020, leading to facility-level stock outs and undermining the scale-up momentum – including disruption of supplies needed for training. Between April 2021 and the beginning of the study period in late 2021, Ghana received two consignments of the product totalling 240,000 units via UNFPA, which helped to improve the immediate outlook for supply in-country, however the medium- to long-term stock status at the time of the study was less certain. Stock issues have been noted as a disruption to the roll out of DMPA-SC in other contexts. [2]

Reflecting these gaps and delays in implementation, OJT HCWs were more likely to report fewer components in their training and dissatisfaction with many elements of their training. This was a stark comparison with the FT HCWs, who were broadly satisfied with the breadth and quality of their two-day formal classroom training, although a few indicated that additional time to practice and written and visual materials would have improved their experience. Furthermore, some significant differences emerged between the OJT and FT groups in their clinical knowledge of the DMPA-SC product and their unprompted recall of the critical SI steps, with OJT cohorts showing significantly poorer results on average. These differences corroborate some HCWs’ fears in the qualitative interviews that the quality of information reaching OJT HCWs was not as comprehensive as that received by the FT cohort. Interestingly, however, there seemed to be no major differences emerging in the quality of overall injectables counselling between FT and OJT HCWs, likely reflecting that both populations had experience in counselling on DMPA-IM, allowing them to accommodate the new injectable option relatively easily.

The fact that confidence and skills gaps in this study emerged specifically around client training on SI is not surprising, given evidence from other studies that HCWs find it challenging to reassure women to overcome their initial fear of SI [5,7,15,17,18]. However, it was reassuring that a small number of entrepreneurial HCWs in both the FT and OJT cohort were already developing their own techniques to try and help reassure women about the SI concept – best practices that have been noted to support women’s confidence to SI in other contexts [17] and ones which could be further shared through peer-learning and supportive supervision at facility level.

It was also interesting that in this study any training (whether FT or OJT) appeared to improve HCW attitudes towards the idea of a self-injectable contraceptive, when compared to untrained HCWs. The fact that no significant differences emerged in attitude statements between FT and OJT HCWs is encouraging, and this likely reflects that being exposed to even some basic information about the size and ease-of-use of DMPA-SC can shift HCW attitudes towards supporting self-injection. However, responses on a few of the attitudes statements in the survey (for example, HCWs not trusting women to remember their re-injection dates) and qualitative examples from KIIs (for example, a few HCWs feeling that SI is may not be suitable for women with low literacy) indicate that some trained HCWs may need more reassurance – either via supportive supervision or through meeting satisfied SI users - that women can and do SI safely. HCWs lacking confidence that SI is possible for all clients is certainly not unique to Ghana and similar concerns have been found in other studies [8], but it could in some cases influence HCWs to limit offering the SI option only to certain groups of clients. Evidence from Ghana and other contexts has indicated that with a supportive HCW and the right visual aids, clients of any educational status can be trained to SI [5,10].

It was notable that in the few facilities in this study where the OJT approach was seen to be working well, this tended to be where the FT HCW was an experienced HCW with good training skills, who embedded regular (for example, twice per year) OJT into their work. The slight differences in length of clinical experience reported by FT and OJT HCWs in the HCW survey suggests that facility in-charges are already bearing the HCWs’ seniority in mind when selecting who to attend the formal trainings, however it may help in future to provide non-binding guidance to facility in-charges on selecting FT HCWs with peer-training experience to maximize the impact of OJT. In addition, the one aspect of training that OJT HCWs evaluated more positively than FT HCWs was post-training follow up, highlighting the skills-strengthening opportunities of working alongside experienced peers and directly with clients on a regular basis. This echoes a finding from studies evaluating HCW acceptability of hybrid and e-learning approaches for DMPA-SC and H-IUD, where aspects such as the flexibility and convenience of being able to learn in situ were highlighted [12,19].

For FT and OJT HCWs in this study who received follow-up supportive supervision or mentorship visits, this was noted as being helpful for retaining knowledge and addressing any gaps, but almost all HCWs (regardless of training type) requested this support should occur more frequently. As several RRTs noted, post-training support could arguably overcome many of the challenges noted with the OJT approach, for example, giving both FT and OJT HCWs the opportunity to practice further under observation, share best practices on SI counselling and training challenges, and address competency gaps arising from any delayed or incomplete OJT. However, while there was certainly appetite to conduct such supportive supervision, at the time of the study most RRT members were not involved in any follow-up support for DMPA-SC with HCWs due to lack of time and funding to conduct site visits. The potential of supportive supervision to optimise alternative training approaches has also been noted in an evaluation of e-learning for DMPA-SC in Uganda and Senegal [12].

Recommendations from this study in 2022 led to GHS designing a structured on-the-job training manual in 2023, which outlined for trained HCWs what materials they would need for OJT trainings at their respective facilities, what OJT content they should train on (including all aspects of DMPA-SC provision including client self-injection, practical sessions, follow up information, and data entry), and encouraging timely delivery of OJT after receiving FT. With a more structured OJT approach in place since 2023, future implementation studies could evaluate the approach more rigorously than was possible at the time of developing this study – including evaluating the hypothesis that these alternative training approaches are more cost-effective than traditional training approaches. Further research in Ghana could also leverage the EASIER protocols, which were published after this study was completed, to investigate further the contextual factors that affect the adoption and implementation of self-injectable contraception throughout health systems [20].

While Ministries of Health across low- and middle-income countries are engaged in the roll out of new contraceptive products such as DMPA-SC, they will inevitably have to explore alternatives to classroom-based in-service training to increase training coverage across their workforce, while saving costs. To date, most studies have documented the effectiveness of classroom-based training models – either within a pilot or, if at scale, cascaded down through levels of the health system or staggered geographically in clusters [2,10]. A few studies have demonstrated the effectiveness of e-learning options at achieving comparable knowledge retention and clinical competency - although this is recommended as an adjunct to, rather than a replacement for, classroom in-service training and/or practicum [12,19]. This study builds upon those insights to describe the challenges of ensuring consistent quality of training when implementing an alternative training model at scale and draws out recommendations to optimise such models through standardisation and supportive supervision.

### Limitations

While this study provided valuable information to inform GHS’ continued scale up of DMPA-SC for self-injection in Ghana, several limitations should be noted:

- At the time of designing this study, there was no structured approach to the OJT provision, beyond a directive to FT HCWs to pass on their knowledge. This made it challenging to design a robust evaluation of the approach. For the FT HCWs, where the study authors had access to their curriculum, training agenda and pre-/post-training tests, it was possible to design the survey to assess their experience against the expected curriculum and their expected post-training knowledge retention. However, without a formal OJT curriculum to compare against, there was no way of knowing how many of the components of the FT curriculum would be transferred, when, and in what format. The only option in that case was to assess the HCWs receiving OJT against the same survey questions as the FT HCWs, hypothesising that if knowledge transfer via OJT was incomplete, they would demonstrate lower levels of correct knowledge retention than FT HCWs.
- The sample for the HCW survey was designed to be representative of only sites with at least one FT HCW in the four study regions (i.e., not nationally representative). The facilities prioritised for in-service training roll out were selected by District Directorates and as such may differ in unknown ways in terms of characteristics from the sites where training has not yet been rolled out. Additionally, the picture of the DMPA-SC roll-out in the four study regions may differ from other regions in Ghana at the time.
- Identifying the geographical and sectoral distribution of the DMPA-SC trained healthcare workers in Ghana to establish a complete sampling frame was challenging. This was partly due to the recent national redistribution of facilities across regional and district boundaries in Ghana, plus lack of visibility over the geographic distribution of trainings conducted by private and social marketing organization partners. The most up-to-date data available on public and private sector DMPA-SC training was used to estimate as complete a sampling frame as possible for the FT providers. However, there was no way of knowing *a priori* how many OJT-eligible colleagues at their facilities would have received OJT – making sample estimates challenging. The best the study team could do was use an estimate of OJT-eligible providers at the site to estimate required sample sizes.
- During quantitative data collection, many site substitutions were necessary, as many facilities originally indicated to have an FT HCW in the sampling frame were found to either have lost the HCW to another site, or sometimes that HCW was on leave or attending a training. Where sampled sites were substituted, these were always switched for another trained site in the same district. However, due to these limitations, the resulting sampling frame (and therefore sample) may not be fully reflective of the population of trained public and private facilities in the four regions.
- When selecting study sites for the survey, the inclusion criteria (i.e., minimum thresholds of DMPA-SC training coverage) used to identify the sites in the sampling frame means that the results may not be representative of regions and districts with lower coverage of DMPA-SC training. It is likely that key outcomes (including consistency of OJT training and HCWs’ accurate knowledge of DMPA-SC) will differ in areas where FT coverage is even lower.
- It is probable that given gaps in responses from the FT population at all sites (only 52 of 64 FT providers were surveyed due to staff unavailability on the day of the visit) and given the lower number of HCWs actually receiving OJT than were OJT-eligible, the sample was underpowered to detect small statistical differences between FT and OJT groups. However, the low probability values from both equality-of-proportion tests and logistic regression adjusted odds ratios, when controlling for clustering by facility, suggest the sample had sufficient power to detect the many large observed differences in key outcomes between FT and OJT HCWs. While years of clinical experience may be hypothesised to be a confounder of any differences in knowledge and skills detected between these groups, as HCWs selected for FT were on average slightly more experienced than those eligible for OJT, sensitivity analyses in the logistic regression models suggest it was not a significant confounder of DMPA-SC knowledge and skills specifically.
- Purposive sampling for qualitative elements ensured that individuals with direct experience of DMPA-SC were sampled, however given the partial roll out of DMPA-SC across geographies and levels of the health system at the time of the study, this approach meant that the resulting sample could be subject to unknown bias. Every effort was made to ensure a range of characteristics in the facilities selected for qualitative data collection to minimise this.
- HCW role plays of injectables counselling and self-injection client training were conducted with enumerators trained to score HCWs against set criteria. It is likely that some element of Hawthorne effect influenced how HCWs acted out these role plays. However, HCWs were reassured that these scores were not a personal test and would be anonymised for analysis to minimise this bias.

## Conclusion

Cascaded in-service OJT approaches, such as the one GHS implemented for DMPA-SC, have been hypothesised to potentially increase training coverage while saving training costs. However, this study’s findings highlight the importance of ensuring that such models include adequate checks and balances to standardise the quality and timeliness of OJT, while simultaneously investing in supportive supervision structures to identify and address any incomplete knowledge and skills transfer among all HCWs, in particular on the challenging area of building client confidence to self-inject. The authors recommended to standardise expectations of the timeliness and content and breadth of OJT; issue optional guidance to facility in-charges to prioritise colleagues with peer-training experience for FT; improve consistency of DMPA-SC stock and associated materials to support counselling (e.g., visual aids, calendars, demonstration models); and improve consistency of post-training supportive supervision to address any residual knowledge and skills gaps and optimise the approach. Following this study, GHS implemented several of these recommendations to strengthen the OJT approach in their ongoing efforts to scale up DMPA-SC across Ghana, including developing a structured OJT manual that was launched in 2023 – a revised approach which could be evaluated in future research.

## Supporting information

S1 Table Theoretical framework outlining behavioural and contextual barriers and enablers of HCWs integrating DMPA-SC, including for self-injection, into routine care. (DOCX)

S1 File Inclusivity checklist. (DOCX)

S2 File StaRI checklist. (DOCX)

S3 File STROBE checklist. (DOCX)
