# Supplementary material for: Consistency of on-the-job training implementation for subcutaneous depot medroxyprogesterone acetate in Ghana and associated healthcare worker knowledge transfer: A cross-sectional mixed-methods study
Source: PLOS Glob Public Health. 2026 Apr 30;6(4):e0004799. doi: 10.1371/journal.pgph.0004799 (PMC13132426; doi:10.1371/journal.pgph.0004799)
Supplement: S1 Table — (DOCX) [file pgph.0004799.s001.docx]

#### S1 Table. Theoretical framework outlining behavioural and contextual barriers and enablers of HCWs integrating DMPA-SC, including for self-injection, into routine care

This framework was developed by the co-authors based on the factors identified in the literature as barriers and enablers of integration of DMPA-SC into routine family planning provision at the time of developing the study protocol (in 2021). It was used to develop the provider survey and key informant interview guide

| Stage in journey | Enablers of SI integration | Barriers to SI integration |
| --- | --- | --- |
| Awareness / perception of DMPA-SC | - Positive perception of DMPA-SC as a product - Belief that DMPA-SC training for self-injection is an investment to save time later (reducing repeat DMPA clients) - Positive colleague perspectives on DMPA-SC - Already a provider of injectable contraceptives | - Loyalty to/comfort with DMPA-IM as a product - Belief that contraception in general is unsuitable for certain client segments who might otherwise benefit from self-injection, e.g. adolescents, new users, low-literacy - Fear of losing community status/belief that providers should always administer healthcare - Concern that self-care means lower quality care - Negative associations with self-injection (e.g. drug use) |
| Training in DMPA-SC | - Good quality, comprehensive training in DMPA-SC including for self-injection - Sufficient time/support to practice administering DMPA-SC and training clients on self-injection | - Lack of or insufficient quality training in DMPA-SC, including for self-injection (e.g. poor quality ‘on-the-job’ training) - Insufficient time/support to practice administering DMPA-SC and training clients on self-injection |
| Integrating DMPA-SC for SI into counselling, provision, and data capture | - Confidence and comfort with DMPA-SC - Manageable staffing and client flow to spend time on comprehensive family planning counselling - Ability to articulate the benefits of SI to clients and reassure clients about concerns or misconceptions - Sufficient DMPA-SC stock - Motivation to counsel on self-injection for repeat DMPA-SC provider-administered and DMPA-IM clients, rather than just once - Recognition/support from facility in-charge/colleagues - Supportive supervision visits/continuous mentoring to improve skills | - Discomfort/lack of confidence providing DMPA-SC - Short-staffing and high client flow leading to time scarcity and reliance on provider-administered injectables, as immediately quicker to provide - Lack of DMPA-SC stock, leading to reliance on DMPA-IM, or just DMPA-SC provider-administration as insufficient stock to send client home with additional doses - Belief that DMPA clients who refused self-injection once will always refuse it in future - Unsupportive facility in-charge/colleagues - Lack of supportive supervision visits/continuous mentoring to improve skills or fill gaps |
| Providing follow on support to DMPA-SC self-injection clients after initial training | - Availability of re-injection aids, e.g. calendars/leaflets, to provide to self-injection clients - Comfort offering follow up calls/visits for self-injection clients with questions - Ability to rely on other health care agents (e.g. mobilisers, community-based distributors) to support self-injection clients in the community | - No re-injection aids, e.g. calendars/leaflets, to provide to self-injection clients - Discomfort handing out phone number/receiving follow up calls from self-injection clients with questions - Self-injection clients living in remote/inaccessible areas, having no access to phones, or being covert users making follow up challenging |
