# Supplementary material for: Consistency of on-the-job training implementation for subcutaneous depot medroxyprogesterone acetate in Ghana and associated healthcare worker knowledge transfer: A cross-sectional mixed-methods study
Source: PLOS Glob Public Health. 2026 Apr 30;6(4):e0004799. doi: 10.1371/journal.pgph.0004799 (PMC13132426; doi:10.1371/journal.pgph.0004799)
Supplement: S3 File — (DOCX) [file pgph.0004799.s004.docx]

STROBE Statement—checklist of items that should be included in reports of observational studies

|  | Item No. | Recommendation | Page  No. | Relevant text from manuscript |
| --- | --- | --- | --- | --- |
| **Title and abstract** | 1 | (*a*) Indicate the study’s design with a commonly used term in the title or the abstract | 1-2 | Title: “a cross-sectional mixed-methods study”  Abstract: “This cross-sectional mixed-methods implementation study” |
|  |  | (*b*) Provide in the abstract an informative and balanced summary of what was done and what was found | 2 | See full Abstract |
| Introduction | | | |  |
| Background/rationale | 2 | Explain the scientific background and rationale for the investigation being reported | 4-6 | See full Introduction section |
| Objectives | 3 | State specific objectives, including any prespecified hypotheses | 6 | Research objectives outlined clearly |
| Methods | | | |  |
| Study design | 4 | Present key elements of study design early in the paper | 8 | See full Materials and Methods section, specifically section on “Research design” |
| Setting | 5 | Describe the setting, locations, and relevant dates, including periods of recruitment, exposure, follow-up, and data collection | 7-13 | See full Materials and Methods section, specifically “Study sites and samples” and “Data collection” sections |
| Participants | 6 | (*a*) *Cross-sectional study*—Give the eligibility criteria, and the sources and methods of selection of participants | 9-11 | 64 sites were sampled via probability proportional to size across 16 districts in four regions (selected for relatively higher DMPA-SC training coverage at time of study).  Study sites and samples: “Within each site, all FT providers and OJT-eligible providers were eligible and invited to be surveyed, including those who were OJT-eligible but untrained, as it was not possible to estimate a priori what percentage of the OJT-eligible population would have actually received OJT.” |
|  |  |  |  |  |
| Variables | 7 | Clearly define all outcomes, exposures, predictors, potential confounders, and effect modifiers. Give diagnostic criteria, if applicable | 8-9 | See “Research outcomes” section within the Materials and methods section |
| Data sources/ measurement | 8* | For each variable of interest, give sources of data and details of methods of assessment (measurement). Describe comparability of assessment methods if there is more than one group | 11-12 | See “Data collection” section, within Material and methods section |
| Bias | 9 | Describe any efforts to address potential sources of bias | 11-12  12-13  33-36 | See “Data collection” section for approaches to minimizing bias within data collection. See “Data analysis” section for details on missing data and sensitivity analyses. See “Limitations” section for descriptions of how efforts to minimize sampling bias were applied |
| Study size | 10 | Explain how the study size was arrived at | 9-11 | See ‘Study sites and samples’ section |

Continued on next page

| Quantitative variables | 11 | Explain how quantitative variables were handled in the analyses. If applicable, describe which groupings were chosen and why | 12-13 | See “Data analysis” section |
| --- | --- | --- | --- | --- |
| Statistical methods | 12 | (*a*) Describe all statistical methods, including those used to control for confounding | 12-13 | See “Data analysis” section |
|  |  | (*b*) Describe any methods used to examine subgroups and interactions | 12-13 | See “Data analysis” section |
|  |  | (*c*) Explain how missing data were addressed | 12-13 | See “Data analysis” section |
|  |  | (*d*) *Cross-sectional study*—If applicable, describe analytical methods taking account of sampling strategy | 12-13 | See “Data analysis” section |
|  |  | (*e*) Describe any sensitivity analyses | 12-13 | See “Data analysis” section |
| Results | | | | |
| Participants | 13* | (a) Report numbers of individuals at each stage of study—eg numbers potentially eligible, examined for eligibility, confirmed eligible, included in the study, completing follow-up, and analysed | 14 | “Participant characteristics” Within the sites, all FP providers were eligible. 81% of total eligible FT providers at the sites participated (52 out of 64). It is unknown how many OJT-eligible HCWs were unavailable on the day of the visit. |
|  |  | (b) Give reasons for non-participation at each stage | N/A | Data on reasons for non-participation was not captured by fieldwork team |
|  |  | (c) Consider use of a flow diagram | N/A | Data on reasons for non-participation was not captured by fieldwork team |
| Descriptive data | 14* | (a) Give characteristics of study participants (eg demographic, clinical, social) and information on exposures and potential confounders | 14 | See “Participant characteristics” section |
|  |  | (b) Indicate number of participants with missing data for each variable of interest | 13 | See “Data analysis” for discussion on missing data |
| Outcome data | 15* | *Cross-sectional study—*Report numbers of outcome events or summary measures | 14-29 | See full Results section |
| Main results | 16 | (*a*) Give unadjusted estimates and, if applicable, confounder-adjusted estimates and their precision (eg, 95% confidence interval). Make clear which confounders were adjusted for and why they were included | 12-13  14-29 | “Data analysis” section outlines that only clustering by facility was adjusted for in analysis. Results section outlines the equality of proportion tests and also adjusted ORs from logistic regression |
|  |  | (*b*) Report category boundaries when continuous variables were categorized | N/A | All variables binary or categorical. |
|  |  | (*c*) If relevant, consider translating estimates of relative risk into absolute risk for a meaningful time period | N/A | No relative risks presented |

| Other analyses | 17 | Report other analyses done—eg analyses of subgroups and interactions, and sensitivity analyses | 12-13 | See “Data analysis” section |
| --- | --- | --- | --- | --- |
| Discussion | | | | |
| Key results | 18 | Summarise key results with reference to study objectives | 29-36 | See “Discussion” and “Conclusion” sections |
| Limitations | 19 | Discuss limitations of the study, taking into account sources of potential bias or imprecision. Discuss both direction and magnitude of any potential bias | 29-36 | See “Limitations” in “Discussion” section |
| Interpretation | 20 | Give a cautious overall interpretation of results considering objectives, limitations, multiplicity of analyses, results from similar studies, and other relevant evidence | 29-36 | See “Discussion” and “Conclusion” sections |
| Generalisability | 21 | Discuss the generalisability (external validity) of the study results | 29-36 | See “Discussion” and “Conclusion” sections |
| Other information | |  | | |
| Funding | 22 | Give the source of funding and the role of the funders for the present study and, if applicable, for the original study on which the present article is based | N/A | Financial statement uploaded as part of submission. PLOS templates advise omitting financial statement from paper for peer-review purposes. |

*Give information separately for cases and controls in case-control studies and, if applicable, for exposed and unexposed groups in cohort and cross-sectional studies.

**Note:** An Explanation and Elaboration article discusses each checklist item and gives methodological background and published examples of transparent reporting. The STROBE checklist is best used in conjunction with this article (freely available on the Web sites of PLoS Medicine at http://www.plosmedicine.org/, Annals of Internal Medicine at http://www.annals.org/, and Epidemiology at http://www.epidem.com/). Information on the STROBE Initiative is available at www.strobe-statement.org.
